# Increased dietary vitamin D was associated with increased circulating vitamin D with no observable adverse effects in adult dogs

**DOI:** 10.3389/fvets.2023.1242851

**Published:** 2023-08-09

**Authors:** Dennis E. Jewell, Kiran S. Panickar

**Affiliations:** ^1^Department of Grain Science and Industry, Kansas State University, Manhattan, KS, United States; ^2^Science and Technology Center, Hill's Pet Nutrition, Topeka, KS, United States

**Keywords:** canine, vitamin D, parathyroid hormone, ionized calcium, health

## Abstract

**Introduction:**

There is no consensus for the optimum concentration of vitamin D, although a minimum concentration of 100 ng/mL (250 nM) of circulating vitamin D, measured as 25(OH) D, has been suggested in order to support optimal health in dogs. Few studies have examined the relationship between dietary vitamin D_3_ (cholecalciferol) intake and the resulting concentrations of circulating 25(OH) D in adult dogs. Recommendations for dog foods for adult maintenance report a safe upper limit of 3,200 IU vitamin D/kg on a dry matter basis. However, these recommendations were not based on studies of adult maintenance requirements. Understanding the relationship between dietary vitamin D and circulating vitamin D is necessary to utilize dietary vitamin D to influence health in dogs.

**Methods:**

Five groups of adult dogs (each *n* = 8) were fed food of approximately 4,000 kcal/kg containing one of the following dry matter concentrations of vitamin D for 6 months: 795.7, 3087.3, 5510.9, 7314.0, and 9992.5 IU/kg. Body weight was recorded at baseline and measured weekly, and daily food intake was recorded. Blood samples were taken at baseline and at the end of the 26-week study period.

**Results:**

There were no clinical signs of vitamin D deficiency or excess. Serum concentrations of creatinine, blood urea nitrogen, albumin, hematocrit, hemoglobin, alkaline phosphatase, phosphorus, total calcium, ionized calcium, and parathyroid hormone were maintained within reference values in all groups. Circulating 25(OH) D increased in all groups except those that consumed food with 795.7 IU/kg vitamin D, and increased in a linear and quadratic fashion in response to dietary vitamin D concentration. All of the dogs fed food with 5510.9 IU/kg vitamin D or above met or exceeded 100 ng/mL (250 nM) circulating 25(OH) D.

**Discussion:**

Dietary vitamin D was positively associated with increased circulating concentrations in concentrations up to 9992.5 IU/kg dry matter, with no observable adverse effects. Consumption of ≥5510.9 IU/kg vitamin D resulted in all dogs with at least the 100 ng/mL (250 nM) circulating concentration.

## 1. Introduction

Vitamin D_3_ (cholecalciferol) has long been known to play important roles in bone health and the regulation of calcium metabolism (1, 2). The last few decades have also established the more widespread functions of vitamin D through the vitamin D receptor present throughout the body (3). Following intake of vitamin D, its hydroxylation in the liver yields 25-hydroxyvitamin D, or 25(OH) D, the major circulating metabolite of vitamin D (4). The serum concentration of 25(OH) D is used as an indicator of vitamin D status (3). In the kidneys, 25(OH) D is further hydroxylated to 1,25-dihydroxyvitamin D, the latter of which is regulated by blood calcium, phosphorus, parathyroid hormone (PTH), and autoregulation (3–5). 1,25-dihydroxyvitamin D acts as a hormone, binding to the vitamin D receptor present in most bodily tissues (3, 4). The activated form of vitamin D receptor acts as a transcription factor, affecting the expression of a wide variety of genes (4).

Sufficient concentrations of vitamin D prevent rickets and osteomalacia. Hypocalcemia, which may result in seizures, can occur in dogs deficient in vitamin D (1, 2). Low serum concentrations of vitamin D in dogs have been observed in a number of conditions, including gastrointestinal disorders, exocrine pancreatic insufficiency, liver disease, immune-mediated diseases, congestive heart failure, several types of cancers, and chronic kidney disease (1, 4–7). Although the causative effect of a lack of vitamin D on many of these disorders is still under investigation, the wide variety of functions underscores the importance of vitamin D. More recently, deficiency of vitamin D in humans was significantly associated with a higher risk of COVID-19 (8), and vitamin D supplementation was found to be one of several factors that decreased the risk of SARS-CoV-2 infection in humans (9).

On the other end of the spectrum, excess consumption of vitamin D can lead to hypercalcemia and detrimental effects on health in dogs (2). Symptoms of excess vitamin D consumption in dogs include weakness, lethargy, anorexia, polyuria, polydipsia, and depression (1, 10). In severe cases, acute renal failure and soft tissue mineralization may occur (1).

Dogs are reliant on dietary sources of vitamin D since they cannot synthesize it in the skin upon exposure to sunlight like other species such as humans (11). The hypothesized minimum concentration of serum vitamin D in dogs is defined as 100 mg/mL (250 nM), based on the concentration of 25(OH) D at which PTH concentration plateaus (12).

The Association of American Feed Control Officials (AAFCO) recommends that dog foods for adult maintenance contain 500–3,000 IU vitamin D/kg (13), and the National Research Council (NRC) recommends 552–3,200 IU vitamin D/kg (14), both on a dry matter basis. Most commercial dog foods are compliant with these guidelines (15). However, the AAFCO and NRC guideline values for safe upper limits were established to prevent impaired ossification in large-breed puppies and were not determined using scientific studies for adult maintenance (5).

Not many studies have examined the relationship between vitamin D intake and resultant concentrations of circulating 25(OH) D in adult dogs. A prior study tested vitamin D supplementation in dogs, most of which were deficient in vitamin D, at 2.3 μg/kg BW^0.75^, which is five times the NRC-recommended amount but within their safe upper limit of 2.6 μg/kg BW^0.75^ (16). At the end of the trial (weeks 9–10), serum 25(OH) D concentrations were significantly higher (but only by 12%) in dogs that received vitamin D supplementation compared with the control group, but the changes from baseline between the two groups were not significant (16).

The aim of this study was to evaluate a range of concentrations of dietary vitamin D over a 26-week feeding period to find those that result in concentrations of circulating vitamin D that are recommended for an appropriate immune response (in excess of 250 nM or 100 ng/mL) in dogs.

## 2. Materials and methods

### 2.1. Ethics statement

All study protocols and this study were reviewed and approved by the Institutional Animal Care and Use Committee, Hill’s Pet Nutrition, Inc., Topeka, KS, United States (Permit Number CP899), and complied with the National Institutes of Health Guide for the Care and Use of Laboratory Animals (17). Dogs were housed in groups, were allowed access to indoor and outdoor runs, and had exposure to natural light that varied with seasonal changes. All dogs were provided with regular opportunities to exercise with access to toys. Dogs were owned by the commercial funders of this research or their affiliates, who gave permission for them to be included in this study. At the conclusion of the study, all dogs were healthy by veterinary examinations and were returned to the Hill’s Pet Nutrition, Inc. colony.

### 2.2. Participants and study design

All dogs were beagles and were determined to be healthy based on the results of an annual physical examination, complete blood count (CBC), serum biochemistries, and urinalysis. The criterion for removal from the study was development of any condition whereby removal would benefit the pet, including polyuria, polydipsia, vomiting, any dog refusing to eat, or inadequate food intake resulting in weight loss greater than 15% of body weight.

Each test food group had 8 dogs (four spayed females, and four neutered males). All dogs had access to electronic feeders for 1 h daily with amounts available for consumption calculated to maintain body weight. Water was available *ad libitum*. Actual daily food intake (g/day) was recorded for each dog, and body weight was recorded each week. Each dog was evaluated daily by animal care technicians for any signs of deficiency or excess including polyuria, polydipsia, lethargy, and depression. At the end of the study, possible toxicity was evaluated through analysis for hypercalcemia and hyperphosphatemia.

Blood was collected following a 20-h fast at baseline and at the end of the 26-week feeding period to evaluate changes in circulating concentration of selected analytes. Analysis of all analytes used the same methods throughout the study and included creatinine, blood urea nitrogen, albumin, hematocrit, hemoglobin, alkaline phosphatase, phosphorus, and calcium (Roche Diagnostics, Cobas 6000 series, c501 module, Indianapolis, IN United States). Ionized calcium, PTH, and vitamin D (25-hydroxy vitamin D) were analyzed by the Michigan State University Veterinary Diagnostic Laboratory (Lansing, MI, United States) (18). Ionized calcium was analyzed using Stat Profile Prime ES Comp Plus analyzer (Nova Biomedical Corporation, Waltham, MA, United States). PTH was evaluated using the Whole PTH (1–84) Specific kit (Scantibodies Laboratory, Inc., Santee, CA, United States), which is an immunoradiometric assay utilizing a polyclonal antibody with a tendency to bind in the N-terminal region and a polyclonal antibody with a tendency to bind in the C-terminal region. The use of these antibodies guarantees that only whole PTH is detected. Validation information is available in Supplementary Data 1. Circulating 25(OH) D was measured in canine sera with a commercially available radioimmunoassay (RIA) kit (25-Hydroxy Vitamin D RIA kit, Immunodiagnostics Systems, Boldon, Tyne & Wear, United Kingdom) that provides reagents necessary for extraction and quantitation of (25)OH D. Validation information is available in Supplementary Data 2.

### 2.3. Study foods

Compositions of the trial foods are shown in Table 1. All foods were complete and balanced, meeting the AAFCO requirements for adult maintenance, with the exception of vitamin D_3_ concentration. Foods with differing concentrations of vitamin D were prepared by Hill’s Pet Nutrition, Inc. and were available in dry form only. Macronutrient composition was determined by a commercial laboratory (Eurofins Scientific, Inc., Des Moines, IA). Proximate analyses were completed using the following techniques: moisture—AOAC 930.15; protein—AOAC 2001.11; fat—AOAC 954.02; ash—AOAC 942.0; fiber—AOAC 962.09 (19). Atwater energy was calculated using the modified Atwater factors as previously described (20). Concentrations of vitamin D_3_ were measured by liquid chromatography/tandem mass spectroscopy (21, 22).

**Table 1:** Composition of trial foods.^.^

	Dietary vitamin D concentrations in study food, IU/kg				
Nutrient	795.7	3087.3	5510.9	7314.0	9992.5
Moisture	8.04	9.71	9.86	9.25	9.52
Protein	24.2	24.5	23.9	24.2	28.0
Fat	14.6	13.3	13.5	13.4	13.3
Ash	5.60	6.01	6.16	5.97	5.60
Calcium	0.70	1.18	1.20	1.14	1.26
Phosphorus	0.55	0.76	0.85	0.85	0.89
Sodium	0.29	0.35	0.36	0.35	0.38
Taurine	0.09	0.11	0.11	0.11	0.11
Crude Fiber	2.0	1.2	1.2	1.1	1.3
Atwater energy, kcal/kg	3,683	3,573	3,572	4,063	4,063
Choline, ppm	3,820	2,740	2,600	2,680	2,430
Vitamin A, IU/kg	9,170	11,700	12,800	12,200	20,300
Vitamin D_3_, IU/kg	731.7	2787.5	4967.5	6637.5	9041.2

All values are g/100 g as-fed unless otherwise indicated.

### 2.4. Statistical analyses

Statistical analyses were performed in SAS, version 9.4 (SAS Institute, Cary, NC). Analysis of the change during the study was completed using PROC GLM with treatment as a fixed variable. Analysis of circulating 25(OH) D was completed using dietary vitamin D concentration as a continuous variable. Values were concluded to be significant if the overall f test was *p* < 0.05 and then the specific mean differences were concluded to be significant at *p* < 0.05.

## 3. Results

### 3.1. Characteristics of dogs in the study

Demographic data for the dogs is shown in Table 2. Dogs in this study ranged in age from 3.2 to 13.3 years. No adverse events were observed in the dogs during the study, and none were removed from the study. All dogs were healthy at the end of the study.

**Table 2:** Demographic data at baseline for healthy adult dogs by vitamin D group (each *n* = 8).

	Dietary vitamin D concentrations in study food, IU/kg				
Parameter	795.7	3087.3	5510.9	7314.0	9992.5
Age, years	9.9 (2.0)	6.1 (2.0)	5.9 (1.8)	6.0 (1.9)	7.3 (1.6)
Sex	4 SF, 4 NM	4 SF, 4 NM	4 SF, 4 NM	4 SF, 4 NM	4 SF, 4 NM
Initial weight, kg	9.73 (1.88)	8.94 (0.88)	11.04 (0.98)	10.74 (0.86)	9.70 (0.98)
Final weight, kg	10.08 (1.82)	9.61 (0.99)	11.15 (1.42)	10.92 (0.76)	10.27 (1.02)
Food intake/day, g	171 (30.0)	176 (26.0)	187 (24.0)	191 (22.6)	172 (18.1)

Data are shown as mean (standard deviation). NM, neutered male; SF, spayed female.

### 3.2. Dietary vitamin D and its influence on circulating analytes

At baseline, the mean serum concentrations of 25(OH) D were above the minimum concentration of 250 nM (Table 3). Individually, 15 (37.5%) dogs had concentrations below this minimum desired concentration at the beginning of the study. All of the dogs throughout the study maintained concentrations within or above the reference range of 109–423 nM. Food intake and body weight were not influenced by the dietary concentrations of vitamin D (Table 2).

**Table 3:** Influence of dietary vitamin D concentrations on selected serum parameters.

		Dietary vitamin D concentrations in study food, IU/kg					
Serum chemistry parameter	Reference range	795.7	3087.3	5510.9	7314.0	9992.5	*p*-value
Creatinine, mg/dL	0.50–1.03^*^						
Initial		0.67 (0.03)	0.68 (0.03)	0.78 (0.03)	0.69 (0.03)	0.66 (0.03)	
Change from baseline		0.05 (0.04)	0.04 (0.03)	0.08 (0.03)	0.07 (0.03)	0.06 (0.03)	0.95
BUN, mg/dL	7.1–25.6^*^						
Initial		13.1 (1.0)	12.7 (1.0)	12.8 (1.0)	11.6 (1.0)	11.5 (1.0)	
Change from baseline		1.8 (1.1)	3.3 (1.1)	2.5 (1.1)	2.4 (1.1)	0.9 (1.1)	0.57
Albumin, mg/dL	2.8–4.0^*^						
Initial		3.62 (0.03)	3.56 (0.03)	3.72 (0.03)	3.76 (0.03)	3.58 (0.03)	
Change from baseline		−0.1 (0.08)	0.20 (0.08)	0.05 (0.08)	0.16 (0.08)	0.18 (0.08)	0.20
Hematocrit, %	44.8–55.6^*^						
Initial		49.5 (1.7)	49.3 (1.7)	51.0 (1.7)	49.0 (1.7)	48.0 (1.7)	
Change from baseline		1.3 (1.7)	0.4 (1.7)	−0.7 (1.7)	1.9 (1.7)	1.2 (1.7)	0.85
Hemoglobin, mg/dL	14.8–19.1^*^						
Initial		17.0 (0.03)	17.2 (0.03)	17.8 (0.03)	16.9 (0.03)	16.7 (0.03)	
Change from baseline		0.55 (0.63)	0.01 (0.63)	−0.26 (0.63)	0.86 (0.63)	0.74 (0.63)	0.67
Alkaline phosphatase, U/L	23–185^*^						
Initial		67.8 (13.4)	100.9 (13.4)	69.1 (13.4)	62.6 (13.4)	64.4 (13.4)	
Change from baseline		−0.1 (8.8)	−2.9 (8.8)	−3.9 (8.8)	−1.4 (8.8)	−4.4 (8.8)	0.99
Phosphorus mg/dL	2.2–5.6^*^						
Initial		4.02 (0.21)	3.45 (0.210)	3.51 (0.210)	3.51 (0.21)	3.67 (0.21)	
Change from baseline		−0.50 (0.24)	0.06 (0.24)	0.46 (0.24)	0.39 (0.24)	0.28 (0.24)	0.06
Calcium, mg/dL	9.1–11.6^*^						
Initial		10.0 (0.1)	9.9 (0.1)	10.2 (0.1)	10.1 (0.1)	10.2 (0.1)	
Change from baseline		0.22 (0.13)	0.53 (0.13)	0.36 (0.13)	0.54 (0.13)	0.18 (0.13)	0.60
Ionized calcium, mM	1.25–1.45^†^						
Initial		1.38 (0.03)	1.38 (0.03)	1.40 (0.03)	1.38 (0.03)	1.37 (0.03)	
Change from baseline		0.01 (0.02)	0.03 (0.02)	0.01 (0.02)	0.02 (0.02)	−0.01 (0.02)	0.51
PTH, pM	1.1–10.6^†^						
Initial		2.03 (0.47)	1.56 (0.47)	1.28 (0.47)	2.81 (0.47)	3.43 (0.46)	
Change from baseline		−0.55 (0.48)	0.26 (0.28)	−0.02 (0.48)	−1.26 (0.48)	−0.46 (0.38)	0.24
25(OH) vitamin D_3_, nM	109–423^†^						
Initial		300 (33.0)	261 (33.5)	264 (37.6)	320 (30.1)	316 (30.6)	
Change from baseline		−45.7 (37.6)^a^	93.2 (37.6)^b^	141.2 (37.6)^b^	110.8 (37.6)^b^	119.9 (37.6)^b^	0.01

All values are means (standard errors). ^*^Normal range from the colony. ^†^Reference ranges from the Michigan State University Veterinary Diagnostic Laboratory (18). BUN, blood urea nitrogen; PTH, parathyroid hormone. ^a,b^Means with different superscripts are different (*p* < 0.05).

**Figure 1:**
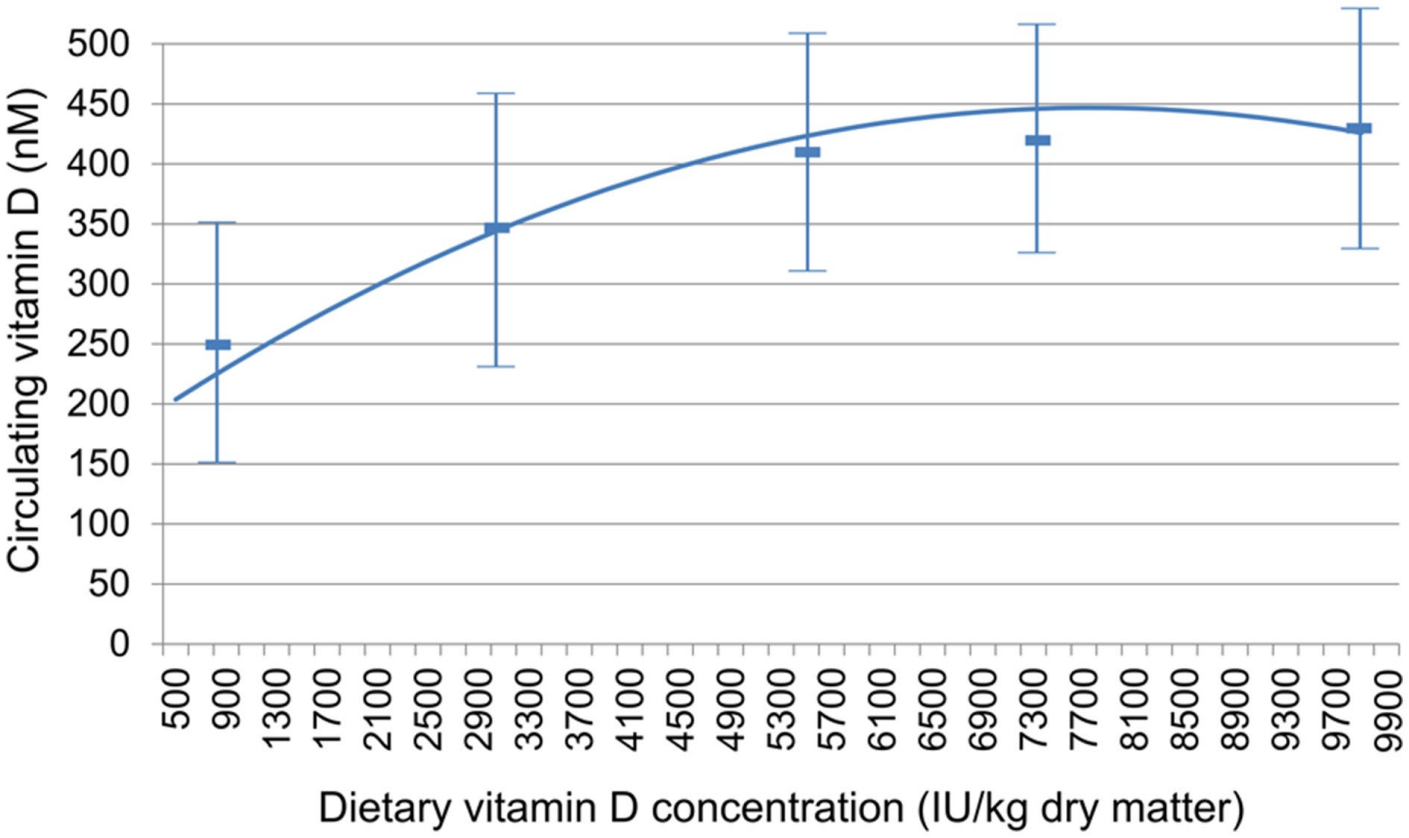
Relationship between dietary vitamin D concentration and circulating vitamin D, measured as 25(OH) D. Data are mean ± standard deviation.

CBC and serum chemistry profiles obtained following a 20-h fast at baseline and at the end of the 26-week feeding period did not significantly change, with the exception of 25(OH) D (Table 3). All of the values except for 25(OH) D remained within the established reference ranges for the colony or diagnostic laboratory. Mean circulating vitamin D concentrations were above the target of 250 nM for all foods at week 26 (Figure 1).

## 4. Discussion

In this study, a range of dietary vitamin D concentrations were tested in order to determine the relationship between dietary intake and circulating 25(OH) D and to also evaluate what intake might result in circulating 25(OH) D concentrations ≥ 250 nM. This concentration was chosen in that although lower concentrations are clearly adequate for maintaining bone health values, concentrations below that level may be suboptimal for non-bone-related vitamin D benefit. The mean serum value of 25(OH) D for the group fed the lowest amount of vitamin D tested (795.7 IU/kg) was 254 nM, a little above the target value. (The decrease from baseline in this group indicates that they were consuming food with higher vitamin D concentrations before the trial.) However, 4 of the dogs in this group had values below this mean and below the suggested optimal concentration. The test foods that contained the three highest vitamin D concentrations resulted in all dogs showing serum 25(OH) D concentrations above 250 nM. The dogs that consumed the food over 5,000 IU/kg vitamin D showed a mean value exceeding 405 nM (162 ng/mL) 25(OH) D, and a population variance (as shown by the standard deviation) that most dogs would achieve the goal of 250 nM.

Excess intake of dietary vitamin D can cause hypercalcemia, hyperphosphatemia, and suppression of PTH (10), none of which were observed in this study. While there were small increases in circulating calcium after the feeding periods for each concentration of vitamin D tested, these were not significant.

No adverse events were observed over this 26-week study period, indicating that the consumption of these concentrations of vitamin D in adult dogs is well tolerated, even though the foods exceed the AAFCO (and NRC) upper limits for vitamin D for adult maintenance. As mentioned, those upper limits were established using data from studies in growing dogs, not adult dogs. In 2016, the AAFCO reduced their safe upper limit from 5,000 to 3,000 IU/kg dry matter to more closely reflect the NRC safe upper limit from 2006, which was based on the results of studies in which Great Dane puppies that consumed the equivalent of 4,000 IU/kg vitamin D had disrupted endochondral ossification (23, 24). Considering the current scientific evidence (16, 23, 24), the guidelines would ideally differentiate between growing and adult dogs and in small and large dogs for vitamin D requirements, as is done for calcium.

In the prior study that tested increased dietary vitamin D and the resultant serum 25(OH) D concentrations, the median dietary vitamin D was 608 IU/kg (after unit conversion) (16), so all of the test foods in the present study contained higher concentrations of dietary vitamin D, as the lowest was 795.7 IU/kg. This may explain the much higher increases in serum 25 (OH) D observed here (ranging from +35% to +53% in the ones that increased) than in the prior study (+12%) (16). Also, as there was no inclusion criterion in the current study for a certain circulating concentration of initial vitamin D, the two studies may reasonably be expected to have different results. In addition, the trial length could have played a role, as the present study was 26 weeks, while the other was only 10 weeks long.

Published values for 25(OH) D in healthy dogs vary. Here, the 25(OH) D concentrations in the dogs at baseline had a mean value of 292 nM (117.1 ng/mL), whereas they were 71.3 ng/mL at baseline in the treatment group in the prior study (16). Because the prior study used dogs with lower concentrations, there may have been specific controlling factors that contributed to that group’s relatively small observed increase in serum 25(OH) D with vitamin D supplementation. Other studies that reported reference values for 25(OH) D in healthy dogs found median serum concentrations of 52.5 ng/mL [range: 14.0–155.6 ng/mL, corresponding to a median of 131.0 nM (range: 34.9–388.3 nM)] (25), 67.9 ng/mL (range: 16.9–349.2 ng/mL) (26), and 68.9 ng/mL (range 9.5–249.2 ng/mL) (12) and mean of 84.90 ± 3.36 ng/mL (27). Since these values are less than the 100 ng/mL suggested as the minimum (12), this indicates that a large number of dogs may benefit from increased dietary vitamin D. However, some of the variation in values among these studies could be due to the use of different assays for circulating vitamin D and a lack of standardization among laboratories. Even within one of the prior studies, 25(OH) D concentrations were lower as measured by high-performance liquid chromatography compared with a chemiluminescence immunoassay, though the results were positively correlated (16).

In addition, sex and reproductive status can affect serum 25(OH) D concentrations in dogs (26), though another study found no significant effects of these parameters (25). In the present study, all dogs were spayed or neutered, and each group had equal numbers of males and females, so reproductive status and sex should not have affected the observed serum 25(OH) D concentrations among the test groups.

The present study has the limitations that the benefit is extrapolated from circulating vitamin D concentration and is therefore hypothetical. More information would be of value: for example, this study and future work would benefit through analysis of 1,25(OH)_2_ D and 24,25(OH)_2_ D as well as fibroblast growth factor 23 (4, 5). Also, changes in other nutrients and their subsequent influence on not only circulating vitamin D but on its biological efficacy were not evaluated in this study but have been shown to be important in understanding the efficacy of vitamin D.

In summary, the results of this study show that foods up to a concentration of 9,992 IU/kg did not confer any adverse effects, while foods that exceed 5,500 IU/kg vitamin D resulted in all dogs with serum 25(OH) D concentrations above the suggested minimum value of 250 nM.

## Data availability statement

The raw data supporting the conclusions of this article will be made available by the authors, without undue reservation upon reasonable request.

## Ethics statement

The animal studies were approved by Institutional Animal Care and Use Committee, Hill’s Pet Nutrition, Inc., Topeka, KS, United States. The studies were conducted in accordance with the local legislation and institutional requirements. Written informed consent was not obtained from the owners for the participation of their animals in this study because Hill’s Pet Nutrition, the study sponsor, owned the animals.

## Author contributions

DJ was responsible for data collection and analysis, wrote the first draft, and edited the manuscript. KP reviewed and edited the manuscript. All authors contributed to the article and approved the submitted version.

## Funding

Hill’s Pet Nutrition, Inc. provided funding for this study. The funder (Hill’s Pet Nutrition, Inc.) provided support in the form of salary for one of the authors (KP), the funder had no role in the design of the study; in the collection, analyses, or interpretation of data; or in the writing of the manuscript.

## Conflict of interest

DJ is a former employee and KP is a current employee of Hill’s Pet Nutrition, Inc., a Colgate-Palmolive Company.

## Publisher’s note

All claims expressed in this article are solely those of the authors and do not necessarily represent those of their affiliated organizations, or those of the publisher, the editors and the reviewers. Any product that may be evaluated in this article, or claim that may be made by its manufacturer, is not guaranteed or endorsed by the publisher.

## References

[1] Clarke KE Hurst EA Mellanby RJ. Vitamin D metabolism and disorders in dogs and cats. J Small Anim Pract. (2021) 62:935–47. doi: 10.1111/jsap.13401, PMID: 34323302

[2] Stockman J Villaverde C Corbee RJ. Calcium, phosphorus, and Vitamin D in dogs and cats: beyond the bones. Vet Clin North Am Small Anim Pract. (2021) 51:623–34. doi: 10.1016/j.cvsm.2021.01.003, PMID: 33653533

[3] Stöcklin E Eggersdorfer M Vitamin D. An essential nutrient with versatile functions in nearly all organs. Int J Vitam Nutr Res. (2013) 83:92–100. doi: 10.1024/0300-9831/a000151, PMID: 24491882

[4] Zafalon RVA Risolia LW Pedrinelli V Vendramini THA Rodrigues RBA Amaral AR. Vitamin D metabolism in dogs and cats and its relation to diseases not associated with bone metabolism. J Anim Physiol Anim Nutr. (2020) 104:322–42. doi: 10.1111/jpn.13259 31803981

[5] Weidner N Verbrugghe A. Current knowledge of vitamin D in dogs. Crit Rev Food Sci Nutr. (2017) 57:3850–9. doi: 10.1080/10408398.2016.1171202, PMID: 27171904

[6] Allison LN Jaffey JA Bradley-Siemens N Tao Z Thompson M Backus RC. Immune function and serum vitamin D in shelter dogs: a case-control study. Vet J. (2020) 261:105477. doi: 10.1016/j.tvjl.2020.105477, PMID: 32741494

[7] Weidner N Woods JP Conlon P Meckling KA Atkinson JL Bayle J. Influence of various factors on circulating 25(OH) vitamin D concentrations in dogs with cancer and healthy dogs. J Vet Intern Med. (2017) 31:1796–803. doi: 10.1111/jvim.14834, PMID: 28941306 PMC5697176

[8] Katz J Yue S Xue W. Increased risk for COVID-19 in patients with vitamin D deficiency. Nutrition. (2021) 84:111106. doi: 10.1016/j.nut.2020.111106, PMID: 33418230 PMC7716744

[9] Flegr J Flegr P Příplatová L. The effects of 105 biological, socioeconomic, behavioral, and environmental factors on the risk of SARS-CoV-2 infection and a severe course of COVID-19: a prospective, explorative cohort study. Biol Methods Protoc. (2022) 7:30. doi: 10.1093/biomethods/bpac030, PMID: 36530561 PMC9750789

[10] Bischoff K Rumbeiha WK. Pet food recalls and pet food contaminants in small animals: an update. Vet Clin North Am Small Anim Pract. (2018) 48:917–31. doi: 10.1016/j.cvsm.2018.07.005 30173926

[11] How KL Hazewinkel HA Mol JA. Dietary vitamin D dependence of cat and dog due to inadequate cutaneous synthesis of vitamin D. Gen Comp Endocrinol. (1994) 96:12–8. doi: 10.1006/gcen.1994.1154 7843559

[12] Selting KA Sharp CR Ringold R Thamm DH Backus R. Serum 25-hydroxyvitamin D concentrations in dogs—correlation with health and cancer risk. Vet Comp Oncol. (2016) 14:295–305. doi: 10.1111/vco.12101, PMID: 25041357

[13] Association of American Feed Control Officials. Association of American Feed Control Officials Official Publication. Washington, DC: Association of American Feed Control Officials (2023).

[14] National Research Council Ad Hoc Committee on Dog and Cat Nutrition. Vitamins In: Nutrient requirements of dogs and cats. Washington, DC: The National Academies Press (2006). 193–245.

[15] Kritikos G Weidner N Atkinson JL Bayle J van Hoek I Verbrugghe A. Quantification of vitamin D3 in commercial dog foods and comparison with Association of American Feed Control Officials recommendations and manufacturer-reported concentrations. J Am Vet Med Assoc. (2018) 252:1521–6. doi: 10.2460/javma.252.12.1521, PMID: 29889635

[16] Young LR Backus RC. Oral vitamin D supplementation at five times the recommended allowance marginally affects serum 25-hydroxyvitamin D concentrations in dogs. J Nutr Sci. (2016) 5:e31. doi: 10.1017/jns.2016.23, PMID: 27547394 PMC4976120

[17] National Research Council Committee Update of the Guide for the Care and Use of Laboratory Animals. The National Academies Collection: Reports funded by National Institutes of Health. Washington (DC): National Academies Press (US), National Academy of Sciences (2011).

[18] Nachreiner RF Refsal KR Petroff B Brudvig J. Endocrinology reference ranges Michigan State University, Lansing, MI: Veterinary Diagnostic Laboratory (2023) Available at: https://cvm.msu.edu/assets/documents/VDL/Endocrinology-Reference-Ranges.pdf (Accessed 30 April 2023).

[19] AOAC International. Official Methods of Analysis. 21st ed. eds. George W. Latimer Jr. Washington, DC: AOAC International (2019).

[20] Hall JA Melendez LD Jewell DE. Using gross energy improves metabolizable energy predictive equations for pet foods whereas undigested protein and fiber content predict stool quality. PLoS One. (2013) 8:e54405. doi: 10.1371/journal.pone.0054405, PMID: 23342151 PMC3544805

[21] Huang M LaLuzerne P Winters D Sullivan D. Measurement of vitamin D in foods and nutritional supplements by liquid chromatography/tandem mass spectrometry. J AOAC Int. (2009) 92:1327–35. doi: 10.1093/jaoac/92.5.1327 19916369

[22] AOAC International. AOAC official method 2011.11: Vitamin D in infant formula and adult/pediatric nutritional formula: Ultra-high-performance liquid chromatography/tandem mass spectrometry. Gaithersburg, MD: AOAC International (2012).

[23] Tryfonidou MA Holl MS Stevenhagen JJ Buurman CJ Deluca HF Oosterlaken-Dijksterhuis MA. Dietary 135-fold cholecalciferol supplementation severely disturbs the endochondral ossification in growing dogs. Domest Anim Endocrinol. (2003) 24:265–85. doi: 10.1016/s0739-7240(03)00018-3, PMID: 12742547

[24] Tryfonidou MA Stevenhagen JJ van den Bemd GJ Oosterlaken-Dijksterhuis MA DeLuca HF Mol JA. Moderate cholecalciferol supplementation depresses intestinal calcium absorption in growing dogs. J Nutr. (2002) 132:2644–50. doi: 10.1093/jn/132.9.2644, PMID: 12221224

[25] Alizadeh K Ahmadi S Sarchahi AA Mohri M. The effects of age, sex, breed, diet, reproductive status and housing condition on the amounts of 25(OH) vitamin D in the serum of healthy dogs: reference values. Vet Med Sci. (2022) 8:2360–6. doi: 10.1002/vms3.943, PMID: 36137283 PMC9677387

[26] Sharp CR Selting KA Ringold R. The effect of diet on serum 25-hydroxyvitamin D concentrations in dogs. BMC Res Notes. (2015) 8:442. doi: 10.1186/s13104-015-1360-0, PMID: 26374201 PMC4570747

[27] Casini L Zago D Cavicchioli E Tomiazzo C. Serum 25-hydroxyvitamin D concentration in Japanese Akita dogs: a survey. Vet Anim Sci. (2020) 10:100139. doi: 10.1016/j.vas.2020.100139 32875143 PMC7451697

