# Supplementary material for: Increased dietary vitamin D was associated with increased circulating vitamin D with no observable adverse effects in adult dogs
Source: Front Vet Sci. 2023 Aug 9;10:1242851. doi: 10.3389/fvets.2023.1242851 (PMC10445235; doi:10.3389/fvets.2023.1242851)
Supplement: Supplementary file 1 [file Data_Sheet_1.docx]

Supplementary Material

Increased dietary vitamin D was associated with increased circulating vitamin D with no observable adverse effects in adult dogs

Dennis E. Jewell^*^, Kiran S. Panickar

*** Correspondence:** Corresponding Author: djewell@ksu.edu

# Supplementary Data 1

The 1-84 PTH or whole PTH kit (Scantibodies Laboratory Inc, Santee, CA, USA) is an immunoradiometric assay (IRMA) utilizing a polyclonal 1-84 PTH antibody with a tendency to bind in the N-terminal region of 1-84 PTH (Label Antibody), and a polyclonal 1-84 PTH antibody with a tendency to bind in the C-terminal region of 1-84 PTH (Capture Antibody). The use of these antibodies guarantees that only whole PTH (CAP) is detected. The Label Antibody is labeled with ^125^I-radioligand. The Capture Antibody is fixed to the tubes. 1-84-PTH or whole PTH (CAP) in patient samples is bound both to the tubes and the Label Antibody. After incubation, ^125^I-antibodies and bound ^125^I-antibody fractions are separated by discarding the supernatant. Simple wash steps reduce the nonspecific binding (NSB) to a minimum for increased precision at the low end of the calibration curve. The concentration of whole PTH (CAP) is directly proportional to the radioactivity bound to the tubes after separation. The concentration of PTH in unknown patient samples and controls is determined by interpolation using a calibration curve. The manufacturer reported no cross-reactivity with PTH (7-84). Per the manufacturer, the functional sensitivity of the assay, which is defined as being the measured concentration by imprecision profile for a coefficient of variation (CV) equal to 20%, was determined to be 0.3 pmol/L. Aliquots of canine serum samples of 0.4 and 14.8 pmol/L were mixed at volume combinations of 9:1, 3:1, 1:1, 1:3, and 1:9 and ran as samples in an assay. Recovery rates, expressed as % observed/expected, for the combinations were 71%, 75%, 88%, 98%, and 99%, respectively. Assay repeatability was assessed with 3 pools of canine serum with mean concentrations of 1.3, 3.5, and 12.8 pmol/L. The respective intraassay % CV for 10 replicates of these pools were 4%, 5%, and 3%. The respective interassay % CVs for 11 replicates of these same pools were 11%, 9%, and 9%.

# Supplementary Data 2

25-hydroxy vitamin D (25(OH) D) was measured in canine sera with a commercially available radioimmunoassay (RIA) kit (Immunodiagnostics Systems, Boldon, Tyne & Wear, NE35 9PD, UK) that provides reagents necessary for extraction and quantitation of the analyte. After the addition of sodium hydroxide and acetonitrile reagents, the serum proteins are precipitated, followed by a centrifugation where 50 μL of the supernatant is incubated with ^125^I-labeled 25(OH) D radioligand and ovine antibody to 25(OH) D. The antibody-bound tracer is separated from the free tracer by a short incubation with anti-sheep IgG cellulose, which is centrifuged and then decanted. The manufacturer reported 100% cross-reactivity with 25-hydroxyvitamin D_3_, 75% cross-reactivity with 25-hydroxyvitamin D_2_, >100% cross-reactivity with 24,25-dihydroxyvitamin D_3_, <0.01% cross-reactivity with cholecalciferol D_3_, and <0.30% cross-reactivity with ergocalciferol (D_2_). Per the manufacturer, the analytical sensitivity of the assay, defined as the calculated concentration corresponding to the mean minus two standard deviations of 10 replicates of the 0 standard was <3 nmol/L. Aliquots of canine serum samples of 33 and 334 nmol/L were mixed at volume combinations of 9:1, 3:1, 1:1, 1:3, and 1:9 and run as samples in an assay. Recovery rates, expressed as % observed/expected, for the combinations were 100%, 98%, 93%, 100%, and 97%, respectively. Aliquots of a canine serum sample containing 279 nmol/L were mixed at a 4:1 rate with solutions containing 0, 25, 64, 160, and 400 nmol/L of vitamin D. The % observed/expected recovery rates of added vitamin D for these mixtures were 92%, 84%, 86%, 95%, and 91%, respectively. Assay repeatability was assessed with 4 pools of canine serum with mean concentrations of 56, 131, 260, and 494 nmol/L. The respective intraassay % coefficients of variation (% CV) for 10 replicates of these pools were 7%, 5%, 11%, and 10%. The respective interassay % CVs for these pools were 5% (n=7), 5% (n=7), 9% (n=7), and 15% (n=6).
